# Do Marriage and Cohabitation Provide Benefits to Health in Mid-Life? The Role of Childhood Selection Mechanisms and Partnership Characteristics Across Countries

**DOI:** 10.1007/s11113-018-9467-3

**Published:** 2018-04-23

**Authors:** Brienna Perelli-Harris, Stefanie Hoherz, Fenaba Addo, Trude Lappegård, Ann Evans, Sharon Sassler, Marta Styrc

**Affiliations:** 1 University of Southampton, Southampton, UK; 2 University of Wisconsin, Madison, WI USA; 3 University of Oslo, Oslo, Norway; 4 Australian National University, Canberra, ACT Australia; 5 Cornell University, Ithaca, NY USA; 6 School of Social Sciences, University of Southampton, Bldg 58, Room 4013, Southampton, SO17 1BJ UK

**Keywords:** Marriage, Cohabitation, Partnership, Health, Cross-national

## Abstract

Extensive research has found that marriage provides health benefits to individuals, particularly in the U.S. The rise of cohabitation, however, raises questions about whether simply being in an intimate co-residential partnership conveys the same health benefits as marriage. Here, we use OLS regression to compare differences between partnered and unpartnered, and cohabiting and married individuals with respect to self-rated health in mid-life, an understudied part of the lifecourse. We pay particular attention to selection mechanisms arising in childhood and characteristics of the partnership. We compare results in five countries with different social, economic, and policy contexts: the U.S. (NLSY), U.K. (UKHLS), Australia (HILDA), Germany (SOEP), and Norway (GGS). Results show that living with a partner is positively associated with self-rated health in mid-life in all countries, but that controlling for children, prior separation, and current socio-economic status eliminates differences in Germany and Norway. Significant differences between cohabitation and marriage are only evident in the U.S. and the U.K., but controlling for childhood background, union duration, and prior union dissolution eliminates partnership differentials. The findings suggest that cohabitation in the U.S. and U.K., both liberal welfare regimes, seems to be very different than in the other countries. The results challenge the assumption that only marriage is beneficial for health.

## Introduction

Extensive research has found that marriage provides health benefits to individuals (e.g., Waite and Gallagher 2002; Wood et al. 2007; Hughes and Waite 2009; Umberson 1992; Williams et al. 2011; Robles et al. 2014; Grundy and Tomassini 2010). Health benefits may accrue due to the protective effects of marriage, which often boosts economic resources (Waite and Gallagher 2002), provides social and emotional support (Ross et al. 1990; Umberson et al. 2010), links individuals to social networks (Umberson and Montez 2010), and encourages greater social control (Umberson et al. 2010). The rise of cohabitation, however, and its similarity to marriage in form and function, raises questions about whether simply being in an intimate co-residential partnership conveys the same health benefits as marriage (Musick and Bumpass 2012; Wu and Hart 2002). Some research indicates that cohabitation is becoming similar to marriage, for example, as a way to start co-residential partnerships and a setting for having and raising children (Perelli-Harris et al. 2012). Cohabiting unions are increasing in duration and less likely to end in marriage (Beaujouan and Ní Bhrolcháin 2011; Heuveline and Timberlake 2004; Wiik and Dommermuth 2011). Indeed, cohabitation is a heterogeneous type of union that includes short-term dating-like relationships, couples who are on their way to marriage, and long-term partnerships indistinguishable from marriage (Hiekel et al. 2014; Perelli-Harris et al. 2014). As a result, cohabiting unions, especially if they are of longer duration and involve childrearing, may provide many of the same advantages to health that marriage does, despite the lack of legal recognition.

Nonetheless, prior studies have found that on average cohabitation and marriage differ along several dimensions. Many studies have found that cohabitation is a less stable family type, even for couples who have had children (Andersson et al. 2017; Musick and Michelmore 2016), which raises questions about whether cohabitation, and particularly cohabitation dissolution, may be detrimental to health and well-being (Tavares and Aassve 2013). Across countries, cohabitors are more likely to dissolve their unions (Galezewska 2016), have lower life satisfaction (Soons and Kalmijn 2009) and lower relationship quality (Wiik et al. 2012). Studies from individual countries, mostly from the U.S., indicate that cohabitors are more likely to be depressed (Brown 2000; Lamb et al. 2003), have slightly worse health (Musick and Bumpass 2012), and higher mortality (Liu and Reczek 2012). In general, cohabitation, especially childbearing within cohabitation, seems to be associated with a pattern of disadvantage (McLanahan 2004; Perelli-Harris et al. 2010) that continues across the lifecourse. One of the key issues, therefore, is to what extent poor health among cohabitors is due to the disadvantages that select individuals into cohabitation, rather than the effect of cohabitation itself.

Here, we focus on whether selection mechanisms in early life select individuals into different partnership types and subsequently produce differential health outcomes in mid-life. Mid-life is typically understudied in family demography, especially cross-nationally. We define mid-life as 40–49 for data reasons, but this age range is also important, because most individuals have entered into adulthood and made decisions about whether to marry, even if they postponed marriage. Most people, especially women, have completed their childbearing, but may be in the middle of childrearing. In mid-life, cumulative disadvantage also begins to take its toll, and health disparities become more pronounced (Pearlin et al. 2005). Thus, mid-life is an important life stage to investigate whether those cohabiting at these ages may have worse health than the married.

Prior research has consistently found that cohabitation is selective of disadvantage, and that cohabitors suffer poor outcomes (Kennedy and Bumpass 2008; Lichter et al. 2014; McLanahan and Percheski 2008). The majority of this research focuses on the U.S.; however, the U.S. may be an outlier (Cherlin 2010; Musick and Michelmore 2016), because patterns of partnership formation and dissolution appear to be diverging by education, while divergence in other countries is less pronounced (Perelli-Harris and Lyons-Amos 2016). One explanation for the strong association between family behaviors and disadvantage may be the welfare state context. Prior research has suggested that welfare states may be important for shaping fertility and family behavior. For example, means-tested benefit regimes may encourage a bi-modal distribution of fertility (Rendall et al. 2009, 2010) or exacerbate poverty among single mothers (Brady and Burroway 2012). The U.S. reliance on means-tested benefits and limited welfare provision may result in a stronger association between cohabitation and disadvantage, subsequently resulting in poor health among cohabitors.

In this paper, we examine whether the health differentials between married and cohabiting individuals found in the U.S. hold in other countries, and to what extent controlling for selection can explain this association. The countries represent different welfare regimes (Esping-Andersen 1990): the United States, the United Kingdom, and Australia are usually classified as liberal welfare states with targeted, means-tested benefits, although Australia tends to provide more generous welfare support; Norway is social-democratic with a universal social policy; and Germany is considered a conservative regime that favors the marital male breadwinner model. The countries also have different approaches to legally recognizing cohabitation: the UK and the US allow legal rights in some policy areas but not others (Barlow 2004; Bowman 2010); Australia and Norway provide cohabitors with many rights similar to married couples (Bowman 2010; Perelli-Harris and Sanchez Gassen 2012); and Germany tends to privilege marriage (Perelli-Harris and Sanchez Gassen 2012). In addition, the countries differ in the degree of selection into cohabitation; for example, the educational gradient associated with partnership formation and dissolution is not consistent across countries (Perelli-Harris and Lyons-Amos 2016). Using harmonized datasets to investigate how the association between partnership type and self-rated health differs across countries will tell us to what extent any benefits to marriage are universal or depend on country context. This study contributes to the literature by examining several underexplored questions: (1) Does being in a partnership convey benefits to health in all the studied countries? Is marriage, compared to cohabitation, associated with better health? (2) Do childhood background characteristics attenuate any positive association between partnership or marriage and health? (3) Does controlling for characteristics of the union reduce differences between cohabitation and marriage? Taken as a whole, the paper provides greater understanding of the meaning and consequences of cohabitation in mid-life across countries.

## Background

### Benefits to Cohabitation and Marriage

Living in an intimate partnership, either marriage or cohabitation, may provide advantages that could directly influence health. By living together, couples can benefit from shared resources, sexual and emotional intimacy, companionship, and daily interaction (Waite 1995). Couples who live together often provide each other with care and monitor each other’s health behaviors, for example, reminding each other to go the doctor or maintain a healthy lifestyle (Musick and Bumpass 2012; Umberson et al. 2010). Through social ties, partners link each other to broader networks, which can instill a sense of kinship and responsibility (Umberson and Montez 2010). Although poor-quality relationships may result in strain and stress (Umberson et al. 2006), in general co-residential relationships provide positive psychosocial benefits by offering social support and providing symbolic meaning to one’s life (Umberson and Montez 2010). Hence, living in a partnership regardless of its type may be what is most important to health.

On the other hand, the official act of marriage may convey unique benefits to health that go beyond simply living with a partner. With a public vow and a legal contract, marriage usually signals a higher commitment between the partners—to family, friends, and strangers, but also to each other (Berrington et al. 2015; Cherlin 2004; Wiik et al. 2009). Married people may have a stronger sense of the long-term prospects of their relationship, since marriage is usually intended for life. Those outside the relationship may find it easier to understand the spouses’ commitment, and therefore provide greater social support (Marcussen 2005). Marriage’s “enforceable trust” (Cherlin 2004) may persuade couples to work harder on their relationships, especially during stressful periods. In addition, marriage may provide a sense of security and well-being. Focus group respondents throughout Europe and Australia mentioned dimensions of marital security that generally did not apply to cohabitation, for example, emotional reassurance, financial stability, security for their children, and the comfort of not being alone in old age (Perelli-Harris et al. 2014). This sense of security may be bolstered by the additional level of legal protection that marriage provides in some countries (Perelli-Harris and Sanchez Gassen 2012). Thus, the higher commitment of marriage may reduce life uncertainty and increase general well-being, which could then have positive effects on health (Liu and Umberson 2008).

### Early Life Conditions

A positive association between marriage and health may not indicate a causal relationship, but instead be due to selection. In this paper, we focus on selection mechanisms that influence partnership choices before entrance into union, in particular parental socio-economic status and family structure in childhood. The experience of childhood adversity may lead to both low-quality adult relationships and future poor health through the accumulation of disadvantage and stress over the life course (Hayward and Gorman 2004; Umberson et al. 2014). In addition, childhood may be a sensitive period during which significant stress or adversity triggers psychological or physiological reactions leading to chronic disease and/or life-long poor health (Haas 2008; Umberson et al. 2014). Controlling for childhood conditions before entrance into adulthood may be sufficient for explaining differences in the association between partnership status and health.

In many countries, father’s low social class and childhood poverty are associated with poor adult health (Haas 2008; Kuh et al. 2004; Luo and Waite 2005). Childhood deprivation may also result in fewer resources and skills in adulthood, which may hamper individuals from finding a suitable marriage partner or achieving the perceived economic bar necessary for marriage (Berrington and Diamond 2000; Oppenheimer 2003; Smock 2000). Parental divorce may also be an important selection mechanism for cohabitation. Those who experienced parental divorce may be jaded with the institution of marriage or not want to risk the financial, social, and emotional costs of divorce (Liefbroer and Elzinga 2012; Perelli-Harris et al. 2017). Parental divorce may also have long-term negative effects on peoples’ well-being (Kuh et al. 2004), which may be one of the underlying reasons why cohabitors have worse health than married individuals.

### Variation in Partnerships: Union Duration, Prior Union Dissolution, and Childbearing

Examining current partnership status alone may not be sufficient for understanding the strength of the couple’s relationship and its benefits to health. Cohabiting couples who live together for a long period may be very similar to married couples, since longer union duration often signals deeper relationship commitment, investments in the relationship such as pooling of resources (Lyngstad et al. 2011), and better relationship quality, which is associated with a range of physical health outcomes (Robles et al. 2014). Staying married and not experiencing union dissolution may also be of primary importance; prior research has found that the experience of divorce can be stressful with long-term ramifications for health (Hughes and Waite 2009). Finally, children can signal investment in a relationship (Berrington et al. 2015; Perelli-Harris et al. 2014) and positively influence future health, since parents may adopt healthier behaviors for the sake of their children (Hank 2010; Read et al. 2011). Thus, these characteristics of the partnership may be very important for explaining the association between partnership type and health in mid-life.

### Differences Across Countries

Cultural, economic, and legal factors have produced differential rates of the decline in marriage and increase in cohabitation, and may result in different associations between marriage and well-being. Policy developments may have exacerbated the increase in cohabitation in some countries, although the increase in cohabitation may also have prompted changes in legislation. Some welfare states recognize cohabitation as an alternative to marriage, providing many of the same rights and responsibilities, for example, similar tax benefits, access to courts upon union dissolution, or parental rights to child custody (Perelli-Harris and Sanchez Gassen 2012). The welfare state may also influence partnership decisions. On the one hand, single mother benefits and tax penalties for low-income married couples may encourage women to stay unmarried in order to maintain their eligibility for benefits (Michelmore 2016). On the other hand, tax incentives that promote a breadwinner model may encourage people to marry. Thus, policies and laws may influence people’s decisions about marriage and cohabitation. Below, we discuss how cultural meanings of marriage, selection effects, and policies could produce a different association between marriage, cohabitation, and health in each context.

Marriage in the U.S. has a special status, especially compared to other countries where cohabitation is often perceived as equivalent to marriage (Cherlin 2010). Although cohabitation has increased rapidly over the past decades, the majority of those born in the 1970s had married by their 40s (Kennedy and Bumpass 2008). At all ages, cohabitation in the U.S. is highly selective of the poor and less educated (Kennedy and Bumpass 2008) and associated with poor relationship quality (Brown and Booth 1996), depression (Brown et al. 2006), and physical violence and abuse (Kenney and McLanahan 2006). A recent study that compares partnership types found that after accounting for unobserved heterogeneity, entrance into marriage results in slightly better health than entrance into cohabitation (Musick and Bumpass 2012). One explanation could be that married spouses have access to health care which is denied partners who are unmarried. For the most part, US law does not recognize cohabitation; no states have passed legislation relating to unmarried partners (Bowman 2010). Welfare state policies, however, tend to privilege low-income single mothers, and single-mother benefits may in fact discourage marriage (Lichter et al. 2004). All in all, the strong association between cohabitation and disadvantage in the U.S., combined with a context that legally and socially favors marriage, may result in a negative association between cohabitation and health. After controlling for background characteristics, however, we expect that the difference in self-rated health for cohabiting and married individuals will disappear.

The situation in the UK is similar to that of the US, although the emphasis on marriage as the utmost ideal is less strident. Since the 1970s, the prevalence and duration of cohabitation in the UK has been increasing rapidly. Around 84% of those married in 2004–07 had previously lived together before marrying, usually for around four years (Beaujouan and Ní Bhrolcháin 2011). Long-term cohabitation, however, is less common; only 10% of cohabiting couples were still together after 10 years; about half of the remainder married, and 40% separated (Beaujouan and Ní Bhrolcháin 2011). Thus, while cohabitation is socially acceptable and the majority of the population perceives few differences between cohabitation and marriage (Duncan and Phillips 2008), marriage is generally considered a more committed union and preferred by most (Berrington et al. 2015). The legal situation in England and Wales still reflects this preference for marriage; cohabiting couples are unable to access family courts upon union dissolution and have to pay inheritance tax when one partner dies (Perelli-Harris and Sanchez Gassen 2012). Given the negative educational gradient for having a birth within cohabitation (Perelli-Harris et al. 2010), the lack of legal protection is disproportionately likely to influence those who are less educated. Single-mother benefits in the UK, on the other hand, may not only discourage marriage, but also co-residential partnerships; qualitative research revealed that women on benefits were aware of how many nights their partner could stay over before losing their benefits (Berrington et al. 2015). Overall, we expect that as in the U.S., cohabitation in the UK will be associated with lower self-rated health, but controlling for childhood background characteristics will eliminate most differences between cohabitation and marriage.

In many ways, Australia has had the same Anglo-Saxon development of family behaviors as the U.S. and U.K., but recently some of the legislative and social developments may have produced differences. As in the U.K. and U.S., the majority of first co-residential unions start with cohabitation (Evans 2013), which is widely accepted (Evans and Gray 2005; Qu and Weston 2008). Nonetheless, qualitative research has continued to demonstrate the importance of marriage, especially as the pinnacle of live-in relationships (Carmichael and Whittaker 2007). Recently, studies have found a weak social selection into marriage; highly educated women are more likely to be married than women with lower levels of education (Evans 2015; Heard 2011). Throughout the 1980s and 90s, lawmakers changed policies to provide cohabiting couples the same rights and responsibilities as married couples. In 2009, the Family Law Act was amended to give couples living together for 2 years or having a child together the same access to the courts in relation to property and spousal maintenance on separation (Family Law Amendment (De facto Financial Matters and Other Measures) Act 2008). Access to government welfare payments, on the other hand, is calculated based on household income, which may discourage some couples from moving in together. Thus, although there is weak selection into cohabitation and a slight social preference for marriage, the legal and social acceptability of cohabitation in Australia leads us to expect few differences in the mid-life health of cohabiting and married individuals.

Cohabitation in Norway developed more rapidly and extensively than in the English-speaking countries. Norway’s social-democratic welfare state, which focuses on gender equality and individual autonomy and regulates cohabitation, may have facilitated the increase (Lappegård and Noack 2015). Among men and women born around 1970, 90% of all co-residential unions started with cohabitation (Wiik and Dommermuth 2011), and almost a quarter of the total population (aged 18–55) are currently cohabiting (Noack et al. 2013). Nearly 90% of unions that eventually have children start with cohabitation (Perelli-Harris et al. 2012). Research has shown that childbearing within cohabitation had a negative educational gradient (Perelli-Harris et al. 2010), but now that more births occur within cohabitation than marriage, selection effects are diminishing. Over the past few decades, the legal system gradually provided cohabitors with similar rights to married couples, particularly those having children together, and more recently those that have been in long-term unions. The focus shifted to provide cohabitors with inheritance rights, but unlike married couples, cohabitors still need to have a will or cohabitation contract to inherit from each other (Perelli-Harris and Sanchez Gassen 2012). Nonetheless, although cohabitation is generally considered equal to marriage, socially and legally, many still prefer marriage, especially as a way of formalizing the commitment of parenthood or expressing the ultimate romantic gesture towards each other (Lappegård and Noack 2015). Thus, we expect that cohabiting and married individuals will be similar, especially with respect to self-rated health, but marriage in Norway is unlikely to disappear anytime soon (Lappegård and Noack 2015).

Finally, in Germany, as in the other countries, cohabitation has also recently increased. Unlike the other countries in this study, however, social policies and taxation law continue to favor marriage over cohabitation; the advantages of tax splitting and sharing the health insurance of the main earner are limited to married couples only (Perelli-Harris and Sanchez Gassen 2012). Moreover, Germany was one of the last countries in Europe to introduce joint parental responsibility for non-marital children. Despite shared institutional and political conditions since reunification in 1990 and the alignment of other family behaviors, such as fertility and divorce, the eastern and western parts of the country still differ considerably with respect to prevalence and meaning of cohabitation (Hiekel et al. 2015; Klärner 2015). Differences are especially apparent for childbearing in cohabitation: of those born in the 1971–73 cohort, by 2009, 31 percent of western German mothers had their first birth out of wedlock while this was the case for 61 percent of eastern German mothers (Kreyenfeld et al. 2011). In both parts of the country, a higher educational level increases the likelihood of being married when the first child is born (Perelli-Harris et al. 2010). People who live together in cohabitation or marriage are also similar for some health behaviors, but differ from those who do not live with their partner or singles. For instance, those living with a partner have a reduced probability of exercising (Rapp and Schneider 2013). Overall, we expect that cohabitation in Germany will be associated with lower self-rated health due to social and legal preferences for marriage. However, because of eastern Germany’s impact, we expect the differences in married and cohabiting individuals’ health to be relatively small and to disappear when controlling for background characteristics.

## Data and Methods

### Data

Our datasets are the only surveys we know of to answer our research questions in these contexts. Four surveys are longitudinal and one is cross-sectional linked to population registers in which all individuals are listed. All have sufficient sample sizes in mid-life, information about childhood conditions, and prior partnership and fertility histories, allowing us to control for union duration and childbearing. We spent considerable time harmonizing the variables and models, although some variables (such as region) remain context specific, and others are not available in all countries (such as race and ethnicity in the U.S., UK, and Australia, which is unavailable in Germany and Norway).

In the U.S., we use the National Longitudinal Survey of Youth 1979 (NLSY79), which follows a representative sample of 12,686 individuals born between 1957 and 1964. The NLSY is the only American survey to have comparable information on early life conditions, health, and partnership histories. In 1979, the survey participants were 14–22 years old. They were interviewed annually through 1994 and biennially since. The health and current partnership data come from surveys conducted in 1998–2006, when the respondents were aged approximately 40–49 and 8416 individuals were still participating in the survey. Unfortunately, this was the only age at which NLSY participants were asked about their health. In order to increase comparability across surveys, we use this age range to define mid-life.

In the UK, we use the UK Household Longitudinal Study (UKHLS), which is a nationally representative household-based longitudinal survey. The survey started in 2009 with approximately 51,000 individuals and is conducted annually. Our sample comes from the fourth wave conducted in 2012/2013 with a total of 47,157 individuals surveyed, but only 11,439 of those were between 40 and 49 years old. In Australia, we use the Household, Income and Labour Dynamics in Australia (HILDA), a nationally representative household-based longitudinal survey. The survey started in 2001 and annually interviewed all adults over 15 years old in the selected households. The sample expanded with a general top-up in 2012, and in 2013 (our analysis year) 13,536 individuals were interviewed. After excluding those who were not between 40 and 49 years old, as well as 239 cases who did not answer the question on self-rated health, our sample comprises 2862 individuals. In Germany, we use the Socio-Economic Panel (SOEP), which is a representative longitudinal study of private households, with all members of the household interviewed annually (from the age of 15). SOEP began in 1984 with 12,290 individuals. Apart from the inclusion of participants from the former East-German state after German reunification, it has had several refreshment samples over its 30-year duration in order to assure national representation. Our sample comes from the 2013 wave that surveyed 24,113 individuals, of which 6977 were between 40 and 49 years old. Finally, in Norway, we use the Generations and Gender Survey (GGS), which is a nationally representative cross-sectional survey of respondents aged 18–79 in 2007 that includes information from the administrative register of 15,114 individuals. After excluding seven cases for not answering the question on self-rated health, 2105 individuals aged between 40 and 49 were included in our sample.

The first rows of Table 1 show the entire sample by whether respondents were in a partnership. The second set of rows is restricted to those in a partnership, which was 65% of the sample in the U.S. (*N *= 5450), 77% in the U.K. (*N *= 8809), 70% in Australia (*N *= 1990), 86% in Norway (*N *= 1535), and 79% in Germany (*N *= 5527).

**Table 1:** Percent and number of those partnered or unpartnered, and married or cohabiting, mean self-rated health, and confidence intervals, men and women aged 40–49 *Source* Own calculations with NLSY79, UKHLS, HILDA, GGS, and SOEP

	US		UK		Australia		Norway		Germany	
	Percent (*n*)	Mean (95% CI)	Percent (*n*)	Mean (95% CI)	Percent (*n*)	Mean (95% CI)	Percent (*n*)	Mean (95% CI)	Percent (*n*)	Mean (95% CI)
Partnered	65% (5450)	3.79 (3.75, 3.83)	77% (8809)	3.38 (3.36, 3.41)	70% (1990)	3.48 (3.43, 3.52)	86% (1535)	3.72 (3.67, 3.77)	79% (5527)	3.51 (3.48, 3.52)
Unpartnered	35% (2951)	3.56 (3.52, 3.60)	23% (2630)	3.11 (3.01, 3.16)	30% (872)	3.28 (3.21, 3.35)	14% (224)	3.54 (2.39, 3.67)	21% (1450)	3.29 (3.24, 3.34)
Married	89% (4874)	3.80 (3.78, 3.83)	82% (7226)	3.43 (3.40, 3.46)	87% (1732)	3.47 (3.43, 3.51)	85% (1303)	3.76 (3.67, 3.79)	89% (4910)	3.52 (3.49, 3.54)
Cohabiting	11% (576)	3.57 (3.49, 3.65)	18% (1583)	3.25 (3.19, 3.31)	13% (258)	3.50 (3.38, 3.62)	15% (232)	3.71 (3.53, 3.82)	11% (617)	3.46 (3.39, 3.53)

### Measures

#### Dependent Variable

Our dependent variable is self-rated health. Self-rated health is associated with current and future physical and mental health conditions; it is recognized as a reliable and valid indicator of general health (Hardy et al. 2014). In all surveys, health is measured with a single question (“In general, would you say your health is”) on a five-level scale with responses: 1= *poor*, 2= *fair*, 3= *good*, 4= *very good*, 5= *excellent*. The responses for all countries were originally in reverse order but were recoded so that higher values denote better health. Because self-rated health has context-specific meanings (Hardy et al. 2014), we do not directly compare measures across countries, but keep all analyses specific to each country.

#### Independent Variables

We primarily chose our independent variables for conceptual reasons and based on prior research, as discussed above (see Table 2 for the distribution of variables according to partnership type). However, to facilitate comparability, we also selected variables that were in all or most of the surveys. In order to account for missing data on independent variables, we followed standardized procedures for multiple imputation using the mi impute command in Stata 13.0. The process predicts values on missing data using an iterative method that bases predictions on random draws from the posterior distributions of parameters observed in the sample (Allison 2001).

**Table 2:** Descriptive overview of differences between cohabiting and married individuals aged 40–49 for the US, UK, Australia, Germany, and Norway

	US		UK		Australia		Germany		Norway	
	COH	MAR	COH	MAR	COH	MAR	COH	MAR	COH	MAR
Age (40–49)	40.9	40.8	43.6	44.1	43.6	44.5	44.4	44.6	43.7	44.6
Mean/SD	1.1	1.0	3.6	3.7	2.9	2.9	2.9	2.8	2.9	2.9
Gender (%)										
Male	55	51	51	49	54	47	52	49	46	45
Female	45	49	49	51	46	53	48	51	54	55
Background										
Geographic residence (%)										
1	18	17	37	34	37	34	24	15	15	20
2	26	29	22	20	63	66	76	85	35	27
3	34	36	10	9	–	–	–	–	24	38
4	22	18	31	37	–	–	–	–	26	15
Respondent’s nativity (%)										
Born in country	96	96	92	85	84	67	89	77	95	93
Born outside country	4	4	8	15	16	33	11	23	5	7
Parents’ nativity (%)										
Both parents native	87	86	69	54	42	49	88	90	94	90
At least one parent foreign	13	14	31	46	58	51	12	10	6	10
Ethnicity^a^ (%)										
Majority within country	72	85	94	80	94	99	–	–	–	–
Minority	28	15	6	20	6	1	–	–	–	–
Childhood selection mechanisms										
Parental separation (%)										
Yes	36	21	28	20	24	15	23	14	10	7
No	64	79	72	80	76	85	77	86	90	93
Mother’s age at birth (%)										
< 20 years	30	14	14	13	–	–	8	9	7	5
21–25 years	39	28	36	35	–	–	38	31	26	29
26–30 years	16	30	32	31	–	–	24	30	26	29
30 + years	15	27	18	21	–	–	30	30	41	37
Mother’s education (%)										
Low	43	30	72	73	56	57	20	31	46	43
Medium	49	59	20	20	29	28	71	58	47	46
High	7	11	8	7	15	15	9	11	7	11
Father’s education (%)										
Low	44	32	62	57	36	39	9	16	44	31
Medium	46	48	29	31	49	43	74	66	48	49
High	11	20	9	12	15	18	17	18	8	20
Mother’s employ. status (%)										
Not employed	48	46	29	34	44	43	21	30	35	33
Employed	52	54	71	66	55	57	79	70	65	67
Father’s occupation (%)										
Not employed	7	5	8	8	8	9	8	6	2	3
Low	48	36	58	50	24	22	23	24	70	63
Medium	24	26	9	10	31	29	41	40	24	29
High	21	33	25	32	37	40	28	30	4	5
Union characteristics										
Union duration	3.8	13.3	11.0	17.6	11.4	16.4	8.2	15.6	13.3	19.3
Mean/SD	4.4	6.4	7.5	6.	8.0	7.4	5.9	8.2	6.2	6.0
Number of children (%)										
No children	26	12	39	27	35	7	39	8	19	5
1	17	18	26	26	20	12	32	19	14	10
2	27	41	24	33	27	43	17	48	34	40
3+	30	29	11	14	18	38	12	25	23	55
Ever separated (%)										
No previous cohab. union	45	80	34	77	41	87	27	40	53	78
Separated or divorced	55	20	66	23	59	13	73	60	47	22
Respondent’s socio-economic background										
Education (%)										
Low	15	7	20	15	34	29	5	7	28	23
Medium	72	63	43	37	39	34	68	61	45	45
High	13	30	37	48	27	37	27	32	27	32
Employment status (%)										
Out of labor force	15	12	11	11	13	12	3	11	9	8
Unemployed	5	2	7	3	3	2	9	4	1	1
Employed	80	86	82	86	84	86	88	85	90	91
Total *N*	576	4874	1583	7226	258	1732	617	4910	232	1303

^a^Ethnicity and race are not included in the Norwegian and German surveys. In Australia, this refers to non-indigenous and indigenous

##### Partnership Type

We present two main comparisons: (1) whether respondents currently live with or without a partner; (2) for those living with a partner, whether they reported being in a cohabiting or marital union.

##### Gender and Age

We include basic control variables for gender and age of respondent, since both have been found to influence self-rated health (Cavallo et al. 2015; Dahlin and Härkönen 2013).

##### Region

Region of residence controls for important contextual factors known to influence cohabitation and health. We chose the most relevant measure of region for each country. In Germany, the East–West divide is particularly important for family formation and health. In eastern Germany, nearly two-thirds of children were born in cohabitation, while in western Germany, only one-third were born in cohabitation (Perelli-Harris et al. 2012). The two regions also have large disparities in health, and even in the relationship between childbearing and self-rated health (Hank 2010). Large regional health differentials also exist across the UK (Newton et al. 2015), and thus we control for four major regions: (1) Scotland, Ireland, North England; (2) Midlands and Wales; (3) South West England; (4) South East England. In Norway, regions also matter for health (Norwegian Directorate of Health 2012), and we control for four regions: (1) Oslo area; (2) East area; (3) South and West; (4) Mid and North. In the U.S., we distinguish between (1) Northeast; (2) North Central; (3) South; and (4) West. In Australia, however, we include a control for differences between rural and urban areas because prior studies have found this to be the most meaningful distinction in the country (ABS 2011; Monnat and Beeler Pickett 2011).

##### Nativity Status, Majority/Minority Race or Ethnicity

In all countries, we include indicators for whether the respondent and the respondent’s parents were born in the country. However, including harmonized variables for race and ethnicity is more difficult, due to the context-specific relevance of these factors. In the U.S., race has consistently been found to be important for partnership status, health, and the relationship between the two (Umberson et al. 2014). Scholars have also found health differentials by race and ethnicity in the UK (Evandrou et al. 2016; ONS 2013), and Australia (ABS 2010). Thus, in the U.S., UK, and Australia, we include a dummy variable for white/non-white.^1^ However, in Norway and Germany, information on race and ethnicity is not collected in any survey or register, due to political sensitivities and a very low proportion of non-white individuals.

##### Childhood Selection Mechanisms

As discussed above, we are particularly interested in the childhood selection mechanisms that select individuals into partnerships or produce poor health outcomes. We focus on two types of mechanisms: family structure in childhood and the socio-economic status of parents. Family structure in childhood includes a dummy variable for whether the respondent lived with both parents at age 14 (U.S.), 15 (Norway), or up to age 16 (U.K., Australia, and Germany), and a categorical variable for mother’s age at respondent’s birth (younger than 20, 20–24, 25–29, and over 30).^2^ Socio-economic status of parents includes both mother’s and father’s education (recoded from context-specific variables into low, medium, and high), mother worked when respondent was 14 or 15, and father’s occupation (low, medium, high, and not employed).

##### Family Formation Experience

We include key family formation variables that reflect the strength of the union and may make cohabiting partnerships more similar to marital partnerships. Union duration is derived from partnership histories. In the NLSY79, the partnership histories were collected prospectively at each wave. In UKHLS, partnership histories were collected retrospectively in wave one in 2009/2010 and updated prospectively. In HILDA, the partnership histories were collected retrospectively at first wave in 2001 and updated in the following waves. In the Norwegian GGS, all information about partnerships was retrieved retrospectively in 2007. In the SOEP, marriage histories and, since 2007, partnership histories were collected retrospectively when respondents entered the survey and updated in subsequent waves. Current union duration and its quadratic were included to allow for non-linear duration dependence, and because it resulted in better model fit than a linear specification. Having experienced a separation was entered as a binary indicator; and number of children distinguished between having no children, one child, two children, and three or more children, which can capture the non-linearity (e.g., J or U-shaped) of the effect of having children on health (Read et al. 2011).

##### Socio-economic Status of Respondent

Because of the strong association between socio-economic status and health, we include respondent’s current level of highest education (low, medium, high) and employment status (employed/unemployed/out of the labor market) as controls in the final models.

### Analytical Approach

We estimate the association between current union status and mid-life health using OLS regression methods, which are standard in studies of self-rated health (e.g., Borgonovi and Pokropek 2016; Heggebø and Elstad 2017, n.d.; Williams et al. 2011). We found nearly identical results using ordered logit models (available on request), but present OLS estimates because they provide the easiest comparison across countries, and categorical or logit models would require arbitrary cut-off points.

To answer the set of research questions outlined in the introduction, we apply a step-wise approach that sequentially adds variables into the OLS regression. We show two sets of models: (1) partnered versus unpartnered (Table 3), and (2) married versus cohabiting (Table 4). Although we could include all three partnership states in the same model (i.e., single, cohabiting, married), we analyze them separately, because we are particularly interested in including union duration as a control in the comparison between cohabitation and marriage. The first model on each table includes partnership status and respondent’s age, gender, respondents’ nativity status, their parents’ nativity status, and whether the respondent was in the majority racial or ethnic group. The second model controls for selection mechanisms from childhood—i.e., parental separation and SES—which can influence future partnership decisions and health. Childhood characteristics are exogenous, because they refer to the time before respondents entered a partnership. In the next models, we add controls to capture the respondent’s experience of family formation throughout adulthood: duration of the current union (not relevant for partnered versus unpartnered analysis), number of children, and experience of union separation. These characteristics are not strictly exogenous and may reflect the pathways through which partnership status and type influences self-rated health. Finally, we include educational attainment and employment status assessed during the same wave as the dependent variable to control for current factors that may influence self-rated health.

**Table 3:** OLS coefficients of self-rated health for partnered versus unpartnered individuals aged 40–49 in the U.S., the U.K., Australia, Germany, and Norway *Source*: Own calculations with NLSY, UKHLS, HILDA, SOEP, GGP

Controls	US	UK	AUS	GER	NOR
(1) Baseline model (+ age, gender, geographical residence, respondent’s and parents’ nativity, majority/minority race or ethnicity^a^)	0.18***	0.25***	0.23***	0.18***	0.20*
	(0.02)	(0.03)	(0.04)	(0.03)	(0.08)
(2) + Parents lived together during childhood and SES of parents (parents’ education, mother worked, father’s occ.)	0.17***	0.23***	0.20***	0.17***	0.16*
	(0.02)	(0.03)	(0.04)	(0.03)	(0.08)
(3) + Number of children	0.14***	0.21***	0.19***	0.16***	0.12
	(0.02)	(0.03)	(0.04)	(0.03)	(0.08)
(4) + Ever experienced separation	0.14***	0.14***	0.14***	0.16***	0.06
	(0.03)	(0.03)	(0.04)	(0.03)	(0.08)
(5) + Current SES respondent (employment status, educ. level)	0.10***	0.13***	0.11***	0.06	0.02
	(0.02)	(0.03)	(0.04)	(0.03)	(0.08)
Observation numbers	8401	11,439	2862	6977	1759

Note: Unpartnered is the reference category

*p < 0.05, **p < 0.01, ***p < 0.001

^a^Ethnicity is a politically sensitive topic and usually not included in surveys in Norway or Germany

**Table 4:** OLS coefficients of self-rated health for married versus cohabiting individuals aged 40–49 in the U.S., the U.K., Australia, Germany, and Norway Source: Own calculations with NLSY, UKHLS, HILDA, SOEP, GGP

Controls	US	UK	AUS	GER	NOR
(1) Baseline model (+ age, gender, geographical residence, respondent’s and parent’s nativity, majority/minority race or ethnicity^a^)	0.23***	0.20***	0.04	0.06	0.06
	(0.04)	(0.04)	(0.06)	(0.04)	(0.08)
(2) + Parents lived together during childhood and SES of parents (parents’ education, mother worked, father’s occ.)	0.13**	0.18***	− 0.01	0.04	0.01
	(0.04)	(0.04)	(0.06)	(0.04)	(0.08)
(3) + Union duration (union duration, union duration squared)	0.08	0.16***	− 0.00	0.04	− 0.00
	(0.05)	(0.04)	(0.06)	(0.04)	(0.08)
(4) + Number of children	0.07	0.15***	− 0.05	0.02	− 0.02
	(0.05)	(0.04)	(0.07)	(0.04)	(0.08)
(5) + Ever experienced separation	0.07	0.08	− 0.05	0.03	− 0.04
	(0.05)	(0.04)	(0.07)	(0.04)	(0.08)
(6) + SES respondent (+ employment status, educ. level)	0.02	0.08	− 0.08	0.02	0.01
	(0.05)	(0.04)	(0.07)	(0.04)	(0.08)
Observation numbers	5450	8809	1990	5527	1535

Note: Cohabiting is the reference category

*p < 0.05, *p < 0.01, ***p < 0.001

^a^Ethnicity is a politically sensitive topic and usually not included in surveys in Norway or Germany

## Results

Table 1 compares the mean self-rated health of men and women by current partnership type in mid-life across all five countries. The descriptive results are weighted to be representative at the population level. The first part of the table compares those currently living with a partner (both married and cohabiting) with those not living with a partner. The percent of those living without a partner ranges from 14% in Norway to 35% in the U.S. In all countries, the mean of the self-rated health of partnered individuals is significantly higher than that of unpartnered individuals. The second part of the table directly compares cohabiting and married individuals. The percent in cohabitation among those living with a partner ranges from about 11% in the U.S. to about 18% in the U.K. The confidence intervals indicate that in the U.K. and the U.S. mean self-rated health scores are higher for married individuals compared to cohabiting individuals. However, from these results, we can already see that mean self-rated health does not differ significantly by partnership type in Australia, Norway, and Germany.

### Partnered Versus Unpartnered

We begin by examining whether any type of co-residential partnership, either cohabiting or marital, is associated with better reports of self-rated health. Table 3 summarizes the results of the Ordinary Least-Squares models for self-rated health in mid-life showing the coefficients for being partnered versus unpartnered at the time of the survey. Row 1 shows the baseline model with controls for age, gender, geographical residence, respondent and parents’ nativity, and majority/minority race or ethnicity. We present pooled models that include both men and women, because interactions by gender and partnership status were not significant in any of the five countries. We immediately see that partnership is significantly associated with self-rated health in all countries (*p* < 0.001 level in all countries except Norway, where *p* < 0.05). Although we cannot directly compare effect sizes across countries because assessments of health are country specific, the baseline effect sizes between partnered and unpartnered are relatively similar in all models. Including controls in the models gradually reduces differences between the partnered and unpartnered until no differences remain for German and Norwegian individuals. Controlling for number of children was particularly important for reducing differentials in Norway, possibly because of the underlying health differentials between those with and without children. In Germany, controlling for current education and employment status reduced differences between single and partnered individuals, reflecting the strong effect of socio-economic status on health. Thus, in Germany and Norway, living with a partner does not have a positive effect on health in mid-life, after accounting for controls.

In the English-speaking countries, however, differences between the partnered and unpartnered remained significant at the 0.001 level, despite a large set of control variables. The size of the coefficients was remarkably similar in the three countries. Given the number of background controls in the models, these findings suggest that being in a partnership may indeed provide positive benefits to health in these countries. Nonetheless, we have not been able to control for a large set of important selection mechanisms from childhood. Thus, we cannot conclude that partnership has a causal effect on self-rated health.

### Cohabiting Versus Married

Table 4 summarizes the results of the Ordinary Least-Squares models for self-rated health, showing the coefficients for being married relative to cohabiting. Again, an interaction term between gender and partnership type was not significant. We immediately see strong differences (significant at the 0.001 level) by partnership type in the U.S. and U.K., countries with similar means-tested benefit systems. These results differ from the other countries, where self-rated health did not vary significantly by partnership type.^3^ In the U.S., married people had significantly higher self-rated health than cohabitors even with basic controls, but the association diminished as additional controls were introduced into the model. As expected, controlling for parental separation and socio-economic status of the respondents’ parents reduced the magnitude of the coefficient substantially. However, it was the duration of the union that eliminated differences between cohabitation and marriage, suggesting that the similarity between the two partnership types increases as the duration becomes more similar.

In the U.K., baseline models with basic demographic controls indicate that married individuals have better health than cohabiting individuals (significant at the 0.001 level). Controlling for childhood background characteristics such as parental separation and SES again reduced differences between cohabitation and marriage. Controlling for union duration and number of children reduced differences further, but adding in the experience of union dissolution eliminated significant differences between cohabitation and marriage. These results suggest that in the U.K., the primary difference in self-rated health between cohabiting and married individuals was due to having experienced separation, which is in line with studies finding that divorce often has long-term effects on health (Hughes and Waite 2009; Liu and Umberson 2008). Interaction terms between partnership type and ever separated were not significant, indicating that the effect of partnership status did not differ by having experienced a prior union. However, a model restricted to only those who had previously experienced separation confirms our results by showing no difference between currently cohabiting and married individuals in any country (available on request). These results suggest that the benefits to marriage are primarily due to long-term marital relationships, but partnership type in second unions does not matter. Hence, in both the U.S. and the U.K., partnership history is very important for explaining differences between cohabitation and marriage, but these differences emerge through having experienced prior separation, not whether a second union is legitimated through marriage.

## Discussion

A large number of studies find that marriage is beneficial for health (Grundy and Tomassini 2010; Liu and Umberson 2008; Umberson et al. 2010), but the increase in cohabiting partnerships raises the possibility that it is not marriage per se that matters, but instead simply living with a partner. Here, we find that the positive association between living in any type of partnership and health in mid-life does seem to be universal across our studied countries. These associations do not seem to differ by gender; partnerships seem to be important for both men and women’s health. Nonetheless, after accounting for a host of family background, demographic, and socio-economic characteristics, the differences in health between those living with and without a partner are reduced considerably. More specifically, in Norway, controlling for selection mechanisms and past partnership and childbearing experiences eliminates significant differences completely, while in Germany, the effect goes away after controlling for employment status and educational level. These results indicate that the positive association between living with a partner and health is confounded by other characteristics in individuals’ lives. In the English-speaking countries, differences between the partnered and unpartnered were still evident after including controls, but we suspect that including factors such as health in childhood, relationships with other family members and friends, and current health behaviors, would reduce differentials completely. Unfortunately, these indicators are not available in our surveys. Because we were unable to account for these potential confounding variables, we are reluctant to say partnerships have a causal relationship with health. In any case, it is important to recognize the heterogeneity of people living on their own, and that much of the association between partnership status and health is due to selection.

With respect to marriage and cohabitation, we again find no differences between men and women, and we only find health differentials in the U.S. and U.K., which have very similar welfare state systems, cultural background, and history of early non-marital childbearing. In these countries, some research has found that cohabitors are more likely to have poor health (Musick and Bumpass 2012); however, most studies only differentiate between the married and unmarried (e.g., Grundy and Tomassini 2010), and thus may underestimate the positive influence of cohabitation. Our study indicates that although baseline models show significant differences between cohabitation and marriage, controlling for selection mechanisms from childhood, as well as childbearing and partnership experiences, eliminates significant differences. These results lead us to formulate two main conclusions.

First, in the U.S. and U.K., cohabitation appears to be a symptom of poverty and difficult conditions in childhood, not a cause of poor health. Prior studies have found that cohabitation in these countries is associated with a pattern of disadvantage that is not as strong in other countries (Perelli-Harris and Lyons-Amos 2016). The strong association between cohabitation and disadvantage may be producing health differentials in America and Britain, which do not appear in other countries. The welfare state system may also be exacerbating the situation, as it appears to do for single mothers (Brady and Burroway 2012). In liberal welfare regimes, state provision of benefits is modest, means-tested, and often stigmatized. These policies may directly or indirectly discourage marriage or even stable cohabiting relationships (Berrington et al. 2015). Limited welfare provision coupled with low income may make it more difficult for individuals to achieve the economic stability preferred for marriage (Edin and Kefalas 2005; Reed 2006). Thus, poor health and cohabitation seem to be part of the package of behaviors associated with increasing inequality (Cherlin et al. 2016). One aspect to keep in mind, however, is that even though these two liberal welfare state regimes are similar in many ways, the health care systems in the two countries differ dramatically: all UK residents are guaranteed access to free health care through the National Healthcare System, but low-income U.S. citizens have more limited access to healthcare. Due to the similarity in the results in the two countries, we think it is unlikely that access to health care itself is producing the health differentials, but instead the lesser generosity of the welfare regimes combined with increasing inequality.

Second, even though we found that selection mechanisms were important for reducing health differentials, the characteristics of the partnership were even more important for eliminating differentials. In the U.S., union duration eliminated significant differences between cohabitation and marriage, indicating that those in long-term cohabiting unions were just as healthy as those in long-term marital unions. In the U.K., prior separation explained why cohabitation was more detrimental to health. Separated or divorced individuals are more likely to cohabit in second-order or higher-order partnerships and they are more likely to have poor health, either due to the long-term effects of separation (Hughes and Waite 2009) or selection, again possibly due to disadvantage or poor health. Prior studies have shown that those who have experienced multiple partnership transitions tend to be disadvantaged (Lichter et al. 2010; Perelli-Harris and Lyons-Amos 2016). Taken together, these findings suggest that cohabitation itself is not necessarily detrimental to health. Cohabiting couples in long-term, committed, first unions appear to be just as healthy as married couples.

In contrast to the U.S. and U.K., individuals in Australia, Norway, and Germany have similar levels of self-rated health regardless of whether they are cohabiting or married in mid-life. This result was expected in Norway, a country with a long history of cohabitation, a focus on gender equal policies, and a movement towards legally equalizing cohabitation and marriage (Perelli-Harris and Sanchez Gassen 2012). However, the results for Germany were not expected, given the German state’s privileging of the marital breadwinner model. Nonetheless, our findings corroborate recent research showing similarities in health-related behavior between cohabiting and married people in Germany, although some of the studies show positive health outcomes, for example, declines in smoking (Klein et al. 2013), and others negative, for example, reduced physical exercise and increased body mass index (Klein et al. 2013; Rapp and Schneider 2013). We also expected that Australia would be more similar to its English-speaking counterparts, given a similar cultural preference for marriage and a positive educational gradient of marriage (Heard 2011). Self-rated health, however, did not differ between cohabiting and married individuals, suggesting that other social and policy effects may be in play. The legal recognition of de facto relationships may reflect a general social acceptance of cohabitation and a reduced selection into marriage that could influence health outcomes in mid-life.

Our study is not without limitations. The study used the best available data in each country to answer the research questions; however, by attempting to harmonize across studies, we are limited to a parsimonious model which may not account for all country-specific characteristics associated with union formation and health. We acknowledge that although cohabitation has increased considerably in these countries, the percent cohabiting at ages 40–49 is still relatively small. Our age range in mid-life was limited to the years in which self-rated health was included in the U.S. survey. Health differentials may be relatively minor for this age range, and further health differentials may emerge as health deteriorates at older ages. Nonetheless, mid-life is often understudied in the health and cohabitation literature, and thus it is important to observe to what extent partnership matters for health at this life stage.

In conclusion, this study challenges some of the fundamental assumptions that partnerships, and marriage in particular, lead to better health. While the basic association between partnership and health was significant in all studied countries, the strength of the association was reduced or eliminated by controlling for childhood selection mechanisms, prior partnerships and childbearing, and current education and employment status. Although the association was still significant after including controls in the English-speaking countries, we suspect that further controls would eliminate the association altogether. Thus, the benefits to partnership seem to be limited once other factors more salient for health are taken into account. Our findings also challenge the notion that only legally sanctioned marriage can produce health benefits. The positive correlation between marriage and health was only evident in the U.S. and U.K., and again, selection and partnership characteristics explained the association. Cohabitation in these English-speaking countries is strongly selective of disadvantage, while it is far less so in the other countries. In general, our findings are important for conceptualizing cohabitation; cohabitation is a very heterogeneous type of partnership, and studies that do not control for the variation in union duration and prior experience with union dissolution may be missing important confounders. Finally, our results suggest that policies, norms, and economic conditions can shape the meaning of cohabitation and the lived experience of cohabitors. Further research is needed to provide a deeper understanding of which specific welfare policies and conditions in the U.S. and U.K. are producing these inequalities.

## Footnotes

1 Non-Indigenous versus Indigenous in Australia.

2 This variable is not available in HILDA.

3 We conducted the same analysis for West Germans only and found no statistically significant differences between cohabiting and married individuals. Unfortunately, our sample size is not large enough to conduct analyses for East Germany separately.
